# Supplementary material for: Biochemical and Cellular Characterization of New Radio-Resistant Cell Lines Reveals a Role of Natural Flavonoids to Bypass Senescence
Source: Int J Mol Sci. 2021 Dec 28;23(1):301. doi: 10.3390/ijms23010301 (PMC8745286; doi:10.3390/ijms23010301)
Supplement: Supplementary file 1 [file ijms-23-00301-s001.zip › ijms-1517226-supplementary.pdf]

Table Figure 5a e 5c.

	DCFH Fluorescence (%)				
	**T0**	**T5**	**T10**	**T15**	*p*-value
**SAOS**	98.86±1.77[a,b,c]	122.24±7.76[a,d]	121.28±8.12[b,d]	121.24±9.00[c]	**< 0,001**
**SAOS400**	98.07±1.66[a,b]	108.44±6.67[a,d]	106.20±6.32 [b,d]	104.50±6.49	**< 0,001**
**HT29**	99.26±0.95[a,b,c]	226.05±92.76[a]	227.79±97.08 [b]	227.79±97.07[c]	**0.001**
**HT500**	98.76±1.06[a,b,c]	118.85±0.56[a,d,e]	120.42±0.63 [b,d]	119.71±0.41[c,e]	**< 0,001**

Values are expressed as mean±SD. For each value, superscript lowercase letters in the same row indicate significant differences among observations.

Table Figure 5b e 5d.

	DHE Fluorescence (%)				
	**T0**	**T5**	**T10**	**T15**	*p*-value
**SAOS**	94.68±4.19[a,b,c]	150.90±7.04[a]	151.34±5.91[b]	151.17±6.03[c]	**<0.001**
**SAOS400**	93.98±4.32[a,b,c]	118.18±7.00[a]	115.23±7.02 [b]	118.02±7.03[c]	**<0.001**
**HT29**	99.40±0.68[a,b,c]	119.41±10.19[a]	122.14±9.03[b,d]	121.10±8.70[c,d]	**<0.001**
**HT500**	98.69±0.92[a,b,c]	107.22±0.18[a,d]	107.02±0.32[b,e]	108.63±0.23[c,d,e]	**<0.001**

Table Figure 6a.

	**Control SAOS**	**Control SAOS400**	**IR SAOS**	**IR SAOS 400**	*p*-value
**SA-βGAL positivity (%)**	11.56±2.34[a,b]	37.86±10.88[a,c]	24.38±3.46[c]	36.81±9.52[b]	**< 0,001**

Table Figure 7a.

	**Control HT29**	**Control HT500**	**IR HT29**	**IR HT500**	*p*-value
**SA-βGAL positivity (%)**	5.00±1.83[a,b]	11.00±4.08[c]	17.00±4.83[a]	22.00±4.08[b,c]	**< 0,001**

Table Figures 8a and 8b.

	Cell Viability (%)			
	**IR**	**F**	**IR+F**	*p*-value
**SAOS**	50.30±2.89^{a}	60.93±2.48^{a,b}	49.66±1.35 ^{b}	**< 0,001**
**SAOS400**	83.24±4.70^{a}	84.28±8.73^{b}	63.92±3.02 ^{a,b}	**0,001**
**HT29**	45.32±0.76^{a}	109.04±2.90^{a,b}	46.02±2.00 ^{b}	**< 0,001**
**HT500**	70.82±2.10^{a,b}	111.87±2.08^{a,c}	65.11±0.69 ^{b,c}	**< 0,001**

Table Figures 8 c-d

	**Control**	**IR**	**F**	**IR + F**	**D**	*p*-value
**Cell Viability (%) SAOS400**	86.92±11.58^{a,b}	78.94±6.06^{c,d}	79.82±9.87^{a,e,f}	38.54±3.71^{a,c,e}	28.41±0.20^{b,d,f}	**< 0,001**
**Cell Viability (%)HT500**	99.68±0.27^{a,b,c,d}	70.04±2.79^{a,e,f,g}	61.01±2.40^{b,e,h,i}	46.33±1.91^{c,f,h,l}	15.47±0.86^{d,g,i,l}	**< 0,001**

Table Figure 8 e-f

	**Control**	**IR**	**Q25**	**IR + Q25**	*p*-value
**Cell Viability (%) SAOS400**	96.26±3.91^{a,b}	70.34±6.92^{a,c,d}	84.29±2.79^{b,c,e}	50.15±5.02^{b,d,e}	**< 0,001**
**Cell Viability (%) HT500**	96.60±2.77 ^{a,b}	72.87±5.15^{a,c,d}	90.88±1.80^{c,e}	60.04±4.16^{b,d,e}	**< 0,001**
